# Books Reviewed

**Published:** 1867-11

**Authors:** 


### Editorial Department.

### Books Reviewed.

The Tree of Life, or Human Degeneracy; its Nature and Remedy, as based on the elevatiug principle of Orthopathy. In two parts. By Isaac Jennings, M. D. New York: Miller, Wood & Co., 15 Laight street, 1867.

Dr. Jennings, although having arrived at the venerable age of four score years, does not allow himself retirement from active pursuits in his profession, but again enters upon the field of medical literature with a new work on "Human Degeneracy," treated according to the orthopathic or "upright, upward tendency of natural law," and which he reviews in both its spiritual and physical aspects. The sections devoted to the spiritual decline of man are a series of religious discussions, which we certainly should not have expected to find in a treatise designed for medical gentlemen, unless they are to serve as a stepping-stone to the more purely medical part of the work. The causes. nature and remedy of the physical decline of the race, the author has made the subject of a candid but rather one-sided discussion. In his therapeutics the writer occupies a position far *advanced* to that of his profession; heretofore we have entertained some confidence in the efficacy of various remedies, such as the opiates, etc., but we are told that hygienic conditions, *bread pills* and *water variously colored*, will effect all that can be hoped for from any drug, cases being cited in which the most painful affections were cured by this bread pill practice. While we are directing attention to some of the inconsistencies of the work, we would most cordially commend the chapters on "Alcoholic Stimulants" to the careful perusal of every reader. The character of Dr. Jennigs is clearly imprinted upon this work, and no one will open its pages but be impressed with the purity and honesty of purpose of the man. The publishers have discharged their task in a most creditable manner, the typographical execution being excellent.

---

Circular No. 7, War Department, Surgeon-General's Office, Washington, D. C., July 1, 1867. A Report on Amputations at the Hip-Joint in Military Surgery.

The feasibility of coxo-femoral amputations in military surgery was placed in a most discouraging and doubtful light by the fearful results obtained during the Crimean, Italian and Schleswig-Holstein Wars, and but few surgeons at the beginning of the Rebellion were prepared to meet the exigency or undertake the responsibility of such an operation, with any hope of success. The interesting and elaborate report prepared by Assistant Surgeon and Br'vt. Lieut. Colonel George A. Otis, in an analytical investigation of all authenticated cases of hip-joint amputations thus far performed, but it is especially designed to examine "in how far the experience acquired in the war of the Rebellion has augmented our data for estimating the value of this operation," as a resource in military surgery. And with a view of determining in how far the experience acquired during the late war has increased our means of judging of the value of coxo-femoral amputations after gun-shot injuries, "this report has been divided into an historical summary, an account of individual cases, a citation of the opinions of surgeons and a discussion of results."

From the historical summary it appears that S. F. Morand, a French surgeon, educated in England, after having carefully studied various methods of disarticulation of the femur upon the cadavar, and reported its successful performance in several instances upon inferior animals, was the first to proclaim the feasibility of this operation about the year 1729, yet not until 1773 do we find the first application of it upon a man crushed between the poles of a wagon, by Perault. The patient survived the operation, being subsequently employed in the capacity of a cook at an inn. From this time until the late war 108 exarticulations have occurred in military surgery, there being ten recoveries, "one after a primary, four after intermediate, and five after secondary operations—a percentage of mortality of 96.66." In civil practice 111 operations are recorded, with 46 recoveries, or a mortality rate of 58.56. Of this number the French report eight recoveries and fifteen deaths; the Germans seven recoveries and six deaths; the Poles four operations, which proved fatal; the British sixteen recoveries and thirty-one deaths, and the Americans fifteen recoveries and nine deaths.

Hip-joint amputations in the war of the Rebellion were performed in fifty-three instances; thirty-four occurring in the service of the United States, and nineteen in that of the Confederate. These operations are described and divided into four categories, "primary, intermediate and secondary amputations, and re-amputations." Under the category of *primary* amputations are classed those which have been performed "in the interval between the reception of the injury and commencement of inflammatory symptoms," which period will rarely exceed twenty-four hours. *Intermediate* amputations are considered those made during the period of inflammatory action. *Secondary* operations comprise those performed during the abatement of inflammation, and "when the lesion has in a measure become local and analagous to chronic disease." *Re-amputations* are those in which "an amputation in the coutinuity had preceded the amputation in the contiguity."

Primary amputations were performed in nineteen instances, the average interval from the time of the reception of the injury and the operation being seven hours. Eleven of the patients succumbed to the direct shock of the operation. three lingered for two and two for eight or ten days. In two instances the patients were known to have recovered, but their subsequent history has uot been traced. Oae has snrvived the operation for four years, enjoying good health. Of the eighteen intermediate amputations not one survived, the patients in all instances being ill-fitted to undergo the operation; exhaustion dependent upon surgical fever, hemorrhage and pyæmia being the chief causes of death, but five succumbing to shock. Nine cases, seven deaths and two recoveries are tabulated under *secondary* amputation. The category of *re-amputations* comprises seven cases, four of which recovered. Of the three fatal cases, two sank in a few hours, the third dying from pyæmia.

After a careful recapitulation of the citations of the operating surgeons and an analytical investigation of all the facts in each individual case, the accessions made to our means of estimating the value of coxo-femoral exarticulations in military surgery, Dr. Otis sums up as follows:

"1st.—We have learned that the primary operation for traumatic causes is not uniformly fatal, as has laterly been taught, and are enabled to define three conditions under which it should be undertaken, while two other conditions in which it may be justifiable are left *sub judice.*

"2d.—Much evidence has been brought to counteract the prevailing doctrine that disarticulation at the hip is an exception to the general rule requiring all amputations deemed indispensable to be performed immediately, the eighteen intermediate amputations performed during the war having all resulted fatally.

"3d.—We have proved that secondary amputations at the hip for necrosis of the whole of the femur or for chronic osteomyelitis following gun-shot injuries. may be performed with as successful results as hip-joint amputations for other pathological causes.

"4th.—It has been shown that when, after amputations in the continuity of the thigh, the stump has become diseased, re-amputations at the hip may be done with comparative safety."

Annexed to this report are chromo-lithographs of the seven successful amputations, and two lithographic plates representing the appearance of bones removed in two successful operations, while throughout the work numerous excellent wood cuts illustrate the appearance of gun-shot fractures of the femur, necessitating their removal.

This and other publications prepared under the direction and patronage of the Surgeon-General, Joseph K. Barnes, reflect great merit upon him and entitle him to the warmest thanks of the profession.

---

A Treatise on Human Physiology, designed for the use of Students and Practitioners of Medicine. By John Dalton, M. D., Professor of Physiology and Microscopic Anatomy in the College of Physicians and Surgeons, New York, etc., etc. Fourth edition, revised and enlarged, with 274 illustrations. Philadelphia: Henry C. Lea, 1867.

The remarkable favor with which this treatise has been received by the profession, both at home and abroad, and which it so justly deserves, renders an extensive notice quite superfluous. The present edition has been subjected to a most thorough revision and all recent discoveries in physiological science incorporated. Especially have the sections on the Physiology of the Nervous System received, as the author says, an entire reconstruction, owing to the advances made in the study of the functions of the gray substance of the Spinal Cord by J. Lockhart Clarke, Esq., and those of the Medulla Oblongata and Trapezium by Dr. John Dean, thus placing our knowledge of the base of the brain and of the spinal cord in a new light. We have no doubt that this work will continue to grow in favor, and will be received by students as the text-book *par excellence* on physiology.

---

Hufland's Art of Prolonging Life. Edited by Erasmus Wilson, F. R. S., author of "A System on Human Anatomy," "Diseases of the Skin," etc. From the last London edition. Philadelphia: Lindsay & Blakiston, 1867.

This is an elegant translation from the German, re-arranged, with the addition of numerous notes. It is not especially designed for the physician, but for the public in general, with a particular regard for young people. Physical happiness and well-being is not unfrequently completely sacrificed, through an unpardonable and at times criminal negligence of these general principles, tending to secure health and prolong life. The recognition of these facts and a desire to fill this vacancy in popular literature, induced Dr. Hufland to publish his researches and observations made in this direction. The wholesome and healthy reflections to which the perusal of this work lead, cannot fail to exert the most beneficial influence, and we could wish to see it distributed broadcast over the country.

This work does not conflict with the medical profession, and macrobiosis must not be confounded with medicine, their objects, means and boundaries being entirely different. While the medical art aims, by corroborative and other remedies, to elevate mankind to the highest degree of strength and perfection, the macrobiotic seeks the means by which to prolong life. The author does not view his subject alone from a medical stand-point, but also introduces the moral element, justly maintaining "that the physical man cannot be separated from his higher moral object; and that man will in vain seek for the one without the other, and that the physical and moral health are as nearly related, as the body and the soul. They flow from the same sources; become blended together; and when united, the result is, *human nature, ennobled and raised to perfection.*"

The age of the world the writer does not consider to have exerted any perceptible influence upon the longevity of man, people not infreqnently attaining the age of the patriarchs, even at the present time, and the remarkable instances of old age which are found recorded in the early history of the world are explained by the chronology of the early ages not being the same as that used at the present time. Hensler, with others, has proved "with the highest probability, that the year until the time of Abraham, consisted of only three months; that it was afterwards extended to eight; and that it was not until the time of Joseph that it was made to consist of twelve."

---

Is it I? A Book for Every Man. By Prof. Horatio R. Storer, M. D.

Stimulated by the success of former works, such as "Why Not? A Book for Every Woman." etc., Dr. Storer has startled the public by turning over his attention to the men, who evidently are anxious to know what has happened to them. He discusses the following points: that it is not good to be alone; marriage as a sanitaty measure, how early in life to be observed; rights of husband, are these rights absolute, or reciptocal with duties; should mere instinct be the rule? Arguments as to divorce, a plea for women, to which is added as appendix, a woman's view of Why Not?

There is a popular element in the work which makes it attractive, and all will read this book as they have the other works by the same author, with pleasure and instruction. He has opened in upon the topics of interior life with a boldness which is truly commendable, and it now appears certain that something of good is to grow out of the effort. He is, however, handling with great freedom, subjects which the early generations of Puritans are supposed to have thought best to let mostly alone and to themselves. Dr. Storer's writings have already called New England Divines to his aid, who are not going to have the latest and most important discoveries in morality, made by a doctor in medicine, "*not by no means.*" Dr. Storer has said some things very well, said some very good things, and some very true things, which few had ever uttered, for the ear of the public, until he led the way.

